# Impact of dietary l-arginine supply during early gestation on myofiber development in newborn pigs exposed to intra-uterine crowding

**DOI:** 10.1186/s40104-017-0188-y

**Published:** 2017-07-01

**Authors:** Johannes Gulmann Madsen, Camilo Pardo, Michael Kreuzer, Giuseppe Bee

**Affiliations:** 1 Agroscope Posieux, la tioleyre 4, 1725 Posieux, Switzerland; 2 ETH Zurich, Institute of Agricultural Sciences, Universitätsstrasse 2, 8092 Zurich, Switzerland

**Keywords:** Dietary supplement, Early gestation, Intra-uterine growth restriction, Myofiber hyperplasia, Neonate, Sow prolificacy

## Abstract

**Background:**

Intra-uterine crowding (IUC) observed in hyperprolific sows impairs myofiber hyperplasia and overall fetal growth. Arginine supplementation (ARG) in gestation diets has been shown to positively affect litter and muscle development. The study objective was to assess whether the effect of ARG on offspring characteristics, with special emphasis on myofiber hyperplasia, differs under IUC conditions from these responses, because in that situation growth retardation is particularly prevalent due to reduced fetal nutrient supply. Unilateral oviduct ligation (OL) was used as a model for an uncrowded and hyperprolificacy (IN) as a model for a crowded intra-uterine environment.

**Methods:**

Five OL and five IN sows were fed a diet supplemented daily with either 43 g l-alanine (Ctrl) or 25 g l-arginine from d 14 to 28 of gestation in a cross-over design (two periods: 5^th^ and 6^th^ parity). At farrowing, two male and two female offspring, respectively, with a low and intermediate birth weight (BtW) were selected. After euthanization, the *Semitendinosus* muscle (STM) was removed and weighed, and the light and dark portions (STM_d_ and STM_l_) were prepared for myofiber histochemistry using ATPase staining and the entire STM for gene expression analysis of myogenesis-related genes using RT-qPCR. In addition, various organs were removed and weighed. Data were analyzed using the MIXED model in SYSTAT.

**Results:**

No effect of either IUC or dietary treatment was found in litter characteristics. Offspring of ARG sows displayed a greater muscle area in STM (*P* < 0.01) as a result of the greater myofiber hyperplasia (*P* < 0.01). The increase was more distinct in the STM_l_ (*P* < 0.05) than in the STM_d_ (*P* = 0.131). Offspring of OL sows were heavier at birth (*P* < 0.01), had a heavier STM (*P* < 0.05), liver (*P* < 0.01) and kidney (*P* < 0.05), but when expressed relative to birth weight, these differences were absent. In addition, IUC had an effect (*P* < 0.05) on the expression of one of the myogenesis-related genes investigated.

**Conclusions:**

Independent from the extent of IUC, ARG improved BtW, muscle and organ weights and myofiber hyperplasia in offspring.

## Background

In the last decade, the selection for high prolificacy in modern sow herds has led to a marked increase in litter sizes. One consistent outcome of this strategy was the increasing number of less vital and less mature low birth weight (L-BtW) piglets [1, 2]. In these piglets, prenatal muscle development is impaired [3, 4] as evidenced by the lower myofiber number and the greater number of myofibers still expressing the fetal myosin heavy chain isoform at birth [4]. Compared with their heavier siblings, the lower prenatal myofiber hyperplasia observed in underprivileged piglets has negative consequences on postnatal growth efficiency [5] and lean meat deposition rate [6, 7].

**Fig. 1:**
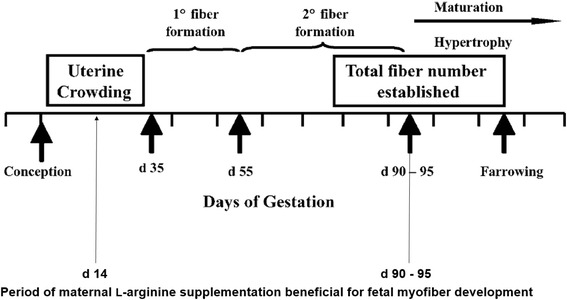
Illustration of myofiber development in offspring during gestation, and the period during which maternal l-arginine supplementation is beneficial to fetal myofiber development. The figure is modified from the review of Foxcroft et al. [47]

The pig muscle develops in a biphasic manner [8] (Fig. 1). In a first wave from d 35-55 of gestation, an initial population of myoblasts fuse to form primary (P) myofibers. From d 55-90 of gestation, these primary myofibers serve as scaffold for the fusion of a second larger population of myoblasts, the so-called secondary (S) myofibers [9, 10]. As opposed to S myofiber development where crowding of the uterus at early gestation appears to be a compromising factor [11], P myofiber number is assumed to be a fixed genetic component, and its development is assumed to be unaffected by conditions occurring in utero [12]. However, a recent study showed that hyperplasia of P myofibers was greater in the *Semitendinosus* muscle (STM) of 75-day-old fetuses originating from sows fed a diet supplemented with l-arginine from d 14-28 of gestation [13]. Because supplementation of l-arginine occurred before the start of P myofiber formation, the authors hypothesized that this effect was an indirect effect. Earlier supplementation of l-arginine from d 0-14 is not advisable because of its detrimental effect on embryonic survival, which is likely due to reduced progesterone secretion [14]. Arginine is a common substrate for nitric oxide and polyamine synthesis [15], both of which are key regulators of angiogenesis and placental growth [16]. Therefore, an increased dietary arginine supply might improve the fetal nutrient supply [17] and ultimately promote myofiber hyperplasia. This mode of action could be interesting especially for offspring from prolific sows suffering from intra-uterine growth retardation (IUGR) due to a crowded intra-uterine environment which impairs placental and, consequently, fetal development.

Based on the aforementioned association between dietary arginine supply, the extent of placental vascularization, the fetal nutrient supply and muscle development, it was hypothesized that supplementing l-arginine to an early gestational diet of the dams would promote hyperplasia leading to an increased number of myofibers in their offspring at birth. A second hypothesis tested the theory of whether l-arginine would be especially efficient in piglets suffering from IUGR. Two sow models were established for the present study, one ‘non-crowded’ and the other ‘relatively crowded’ simulated by intact (IN) prolific sows. The non-crowded intra-uterine environment was mimicked by using unilaterally oviduct ligated (OL) sows. In these sows, oocytes ovulated from the ovary ipsilateral to the ligated oviduct are prevented from being fertilized and entering the uterus. Therefore, the number of embryos and the extent of crowding in utero is markedly reduced in OL compared with IN sows [13, 17].

## Methods

### Animals and dietary treatments

The study was conducted as a 2 × 2 crossover design (two periods for each sow in their 5^th^ and 6^th^ parity). It involved five OL sows originating from a previous experiment of Pardo et al. [18] and five prolific IN sows. The five IN sows were siblings to the five OL sows. Except from d 14 to 28 of gestation, the sows were reared from mating to farrowing with other multiparous sows in group pens equipped with an automatic feeder (Compident, Model 2000, Schauer, Prambachkirchen, Austria) and were offered 2.8 kg of a standard gestation diet daily (Table 1). From d 14 to 28 of gestation, the sows were kept in individual pens. They were randomly allotted to receive daily either 25 g l-arginine HCl (ARG), an amount based on the previous findings of Bérard and Bee [13], or 43 g l-alanine (Ctrl) both as top dressing for 14 d. Alanine was used to compensate for the increased amount of nitrogen in the ARG treatment making the two dietary treatments isonitrogenous.

**Table 1:** Ingredients and calculated nutrient composition of the gestation diet^1^

Item	Gestation diet
Ingredients, % as-fed	
Barley	25.0
Oat	20.0
Dried sugar beet pulp	14.3
Wheat bran	10.0
Soybean meal	5.7
Dried apple pomace	5.6
Dried whole maize plant	5.0
Animal fat (70% lard and 30 % tallow)	3.0
Linseed meal	2.0
Potato protein	2.0
Rapeseed meal	2.0
Molasses	2.0
Dicalcium phosphate	1.16
Calcium carbonate	0.75
Sodium chloride	0.56
Pellan^2^	0.40
Vitamin-mineral premix^3^	0.40
Amino acids^4^, %	
l-lysine HCl	0.08
l-threonine	0.06
Calculated composition, % DM	
Dry matter, % of wet weight	88.35
Total ash	7.20
Ether extract	6.80
Crude protein	15.10
Digestible energy, MJ/kg DM	13.70

^1^In the ARG group, the pellets were top dressed with 25 g l-arginine HCl/(sow·d), and in the Ctrl group, the pellets were top dressed with 43 g l-alanine/(sow·d)

^2^Binder that aids in pellet formation (Mikro-Technik, GmbH & Co. KG, Germany)

^3^Contains: vitamin A: 2,000,000 U/kg; vitamin D_3_: 200,000 U/kg; vitamin E: 10 g/kg; I: 137.4 mg/kg; Mn: 5 g/kg; Cu: 1.75 g/kg; Zn: 1.4 g/kg; Se: 50 mg/kg

^4^Calculated amino acid composition, g/kg DM: alanine: 6.42; arginine: 8.51; aspartate plus asparagine: 12.70; cystine: 3.15; glutamate plus glutamine: 27.50; glycine: 6.54; histidine: 3.42; isoleucine: 5.74; leucine: 10.72; lysine: 7.90; methionine: 2.46; phenylalanine: 7.00; proline: 9.59; serine: 6.73; threonine: 6.30; tryptophan: 1.83; tyrosine: 5.03; valine: 7.38

### Data and tissue sample collection at farrowing

At the end of farrowing, litter characteristics including the number of piglets born alive and stillborn and their individual body weights were recorded. At the day of farrowing, two female and two male piglets per sow per parity were intentionally sacrificed, one with the lowest BtW (L: average ± standard deviation (SD): 1.24 ± 0.30 kg) and one with intermediate BtW (M: average ± STD: 1.49 ± 0.26 kg). Thus, the aim was to include a total of 80 piglets in the study. However, due to unforeseen events at farrowing, the final number was only 70 piglets, which were balanced accordingly between the extent of crowding (38 vs. 32, IN and OL), dietary treatment (36 vs. 34, Ctrl and ARG), BtW (33 vs. 37, L and M), sex (34 vs. 36, male and female), and parity (33 vs. 37, 5^th^ and 6^th^). Pigs with a BtW of < 800 g were considered runts and were not included in the study. The selected piglets were anaesthetized using an isoflurane-oxygen mixture [4% vol/vol] and subsequently euthanized by exsanguination. Subsequently, the complete *Semitendinosus* muscle (STM) and *Psoas major* muscle (PM) were removed and weighed. The STM was split into the light and dark portions (STM_l_ and STM_d_). A section from each portion was removed from the middle of the muscle, snap-frozen in 2-methylbutane cooled in liquid nitrogen and subsequently stored at −80^o^C until histochemical analysis was performed. Subsequently, spleen, kidneys, heart, lungs, liver and brain were removed and weighed.

### Histochemical analysis of the semitendinosus muscle

The P and S myofibers were differentiated histochemically using the protocol described previously by Bérard et al. [4]. Briefly, 10 μm cross-sections of the STM_d_ and STM_l_ were prepared and stained for the determination of myofibrillar ATPase activity after acid (pH 4.5) or alkaline (pH 10.3) preincubation. In the STM_d_, the P myofibers stain dark, and the S myofibers light, using the acid preincubation condition, whereas the opposite occurs after basic preincubation. This differentiation was possible in the STM_d_, whereas, in accordance with observations of the study by Bérard et al. [4], P and S myofibers could not be differentiated in the STM_l_ of newborn pigs using this mATPase histochemistry assay. A sectional cut of the entire muscle area was stained using anti-slow myosin heavy chain monoclonal antibody (Novocastra lyophilized mouse monoclonal antibody myosin heavy chain (NCL-MHCs) diluted 1:20 in ultrapure water; Novocastra, Newcastle, UK). This allowed a clear determination of the STM_d_ and STM_l_ cross-sectional areas.

The number of P myofibers were determined in the mATPase sections after acid pre-incubation, where 750 P myofibers were counted in an area of 0.89 mm^2^. The number of P myofibers, counted in the selected area, and the cross-sectional area of the STM_d_ was used to estimate the total number of P myofibers. The number of S myofibers was determined in mATPase sections after alkaline preincubation. Four images were taken and analyzed using the analySIS software 5.0 (Soft Imagine System, Olympus Soft Imaging Solutions GmbH, Münster, Germany). At least 33 P myofibers were selected, and all the surrounding S myofibers were counted (> 1000 S myofibers). This permitted estimating the total S myofiber number as well as calculating the S:P myofiber ratio. The total number of myofibers (TNF) in the STM_d_ was calculated from the respective estimated total number of P and S myofibers. In the STM_l_, the number of myofibers was determined in mATPase sections after acid preincubation, where > 3000 myofibers were counted in an area of 1.47 mm^2^. The number of counted myofibers, the according measurement area (1.47 mm^2^) and the cross-sectional area of the STM_l_ were used to estimate TNF.

### Gene expression analysis of myogensis-related genes in the *Semitendinosus* muscle

Of the 70 piglets, samples from five individuals (1 L-BtW and 1 M-BtW male from ARG-IN sows, 2 L-BtW females from ALA-IN sows and 1 L-BtW male from ALA-IN sow) were of too poor quality for analysis of gene expression, thus in a total of 65 piglets, total mRNA was extracted from 50 mg STM by the phenol-chloroform extraction protocol involving homogenization with IKA® T10 basic Ultra-Turrax® (IKA, Staufen, Germany) and phase separation with peqGOLD TriFast^TM^ (Peqlab, Erlangen, Germany). Centrifugation steps were carried out at 4 °C and 12,000 × *g*. Tissue was cut into pieces of 5 mg and homogenized for 20 s in 1 mL of TriFast TM reagent. After 5 min of incubation at room temperature, 200 μL of chloroform was added, shaken for 15 s and incubated at room temperature for 10 min. Subsequently, phases were separated by centrifugation, and the supernatant containing the mRNA was carefully removed and mixed with 500 μL of isopropanol, precooled at 4 °C. Following a 15 min incubation on ice, the tube was centrifuged, the supernatant discarded and the pellet washed in precooled (4 °C) 500 μL of ethanol. After a last centrifugation step at 7,500 × *g* for 8 min, the ethanol was discarded and the pellet was dried at room temperature. Finally, the mRNA was resuspended in 100 μL nuclease-free water under agitation at 55 °C. Quality and concentrations of sample mRNA were measured with a NanoDrop® ND-1000 (Thermo Scientific, Waltham, Massachusetts, USA). Primers for both target and reference genes are listed in Table 2. All primers were designed with Primer3 v.4.0.0 (http://primer3.wi.mit.edu/), tested for dimerization with OligoAnalyzer® v.3.1 (Integrated DNA Technologies, Coralville, Iowa, USA), and subsequently purchased in SePOP quality dissolved in water (Eurogentec, Seraing, Belgium). Reverse transcription of complementary DNA (cDNA) was performed with ImProm-II^TM^ Reverse Transcription System (Promega, Dübendorf, Switzerland). First, random hexamer primers were annealed to the mRNA (5 μL of DNAse-treated mRNA with 1 μL of random hexamers, incubation at 70 °C for 5 min. The mRNA was then subjected to the reverse transcription (using 20 μL reaction solutions with 4 μL of 5× buffer, 2.4 μL of 25 mmol/L MgCl_2_, 1 μL of 10 mmol/L dNTPs, 1 μL of reverse transcriptase and 5.6 μL nuclease-free water, according to manufacturer’s protocol). Finally, the freshly synthesized cDNA was diluted 12.5 × with nuclease-free water and stored at 4 °C until further use. Real time-qPCR was carried in 20 μL reaction solutions consisting of 10 μL of 2 × KAPA^TM^ SYBR® FAST Master Mix (Kapa Biosystems, Woburn, Massachusetts, USA), 4.2 μL of water, 0.4 μL 10 μmol/L of both forward and reverse primers and 5 μL of cDNA sample. Amplification was carried out in a Rotor-Gene 6000 (Corbett Life Sciences, Sydney, Australia) under the following conditions: A 3 min hold at 95 °C followed by 40 cycles with 10 s of denaturation at 95 °C, 10 s of annealing at 60 °C: Insulin-like growth factor 2 (*IGF2*), Insulin-like growth factor binding protein 5 (*IGFBP5*), Myogenic factor 6 (*MYF6*), Myogenic differentiation 1 (*MYOD*), Protein kinase, AMP-activated, alpha 2 catalytic subunit (*PRKAA2*), TATA box binding protein (*TBP*), 20 s at 60 °C: Myogenin (*MYOG*) or 20 s at 62 °C: Myostatin (*MSTN*), Myogenic factor 5 (*MYF5*), Ribosomal protein L4 (*RPL4*) and 20 s extension time at 72 °C with the last 4 s being used for fluorescence measurements. The cycling was followed by acquisition of a melt curve (from 72 °C to 95 °C, in increments of 1 °C each 5 s) to control the specificity of the PCR product. Owing to the large number of samples, it was decided to amplify all samples for a given gene in one run. Two technical replicates were performed in two further runs (rather than measuring technical replicates in the same run). To reduce inter-run variation, the reactions in all three runs were prepared with the same mastermix.

**Table 2:** Forward and reverse primers of myogenesis-related genes and reference genes^1^

	Accession no.	Forward primer (from 5’ to 3’)	Reverse primer (from 5’ to 3’)
Gene			
*IGF2*	NM_213883	TGGCATCGTGGAAGAGTG	AGGTGTCATAGCGGAAGAAC
*IGFBP5*	U41340	GTGTACCTGCCCAACTGTGA	AAGCTGTGGCACTGGAAGTC
*MSTN*	NM_214435	CCCGTCAAGACTCCTACAACA	CACATCAATGCTCTGCCAA
*MYF5*	Y17154	CCTGAATGCAACAGCCCT	CGGAGTTGCTGATCCGAT
*MYF6*	NM_001244672	CGCCATCAACTACATCGAGAGGT	ATCACGAGCCCCCTGGAAT
*MYOD1*	NM_001002824	GGTGACTCAGACGCATCCA	ATAGGTGCCGTCGTAGCAGT
*MYOG*	NM_001012406	CAACCAGGAGGAGCGAGAC	GAGGTGAGGGAGTGCAGATT
*PRKAA2*	NM_214266	CCCCTGAAACGAGCAACTATC	CACACTTCTTTCACAGCCTCAT
Reference genes			
*RPL4*	DQ845176	CAAGAGTAACTACAACCTTC	GAACTCTACGATGAATCTTC
*TBP*	DQ178129	GATGGACGTTCGGTTTAGG	AGCAGCACAGTACGAGCAA

^1^ *IGF2* Insulin-like growth factor 2, *IGFBP5* Insulin-like growth factor binding protein 5, *MSTN* Myostatin, *MYF5* myogenic factor 5, *MYF6* myogenic factor 6, *MYOD1* Myogenic differentiation 1, *MYOG* Myogenin (myogenic factor 4), *PRKAA2* Protein kinase AMP-activated, alpha 2 catalytic subunit, *RPL4* (LOC100038029) Ribosomal protein L4, *TBP* TATA box binding protein

The modified formula by Livak and Schmittgen [19] suitable for multiple reference genes was applied to gene expression data, and the relative quantification was performed with the qBase+ software (Biogazelle NV, Zwijnaarde, Belgium) [20].

### Statistical analysis

Data were analyzed using the MIXED procedure of SYSTAT version 13 (Systat Software, San Jose, CA). All data were tested for normality of residuals. For litter traits, data were analyzed including IUC and dietary treatment (DIET), IUC × DIET interaction, parity, and boar as fixed effect, offspring as random effect, and sow as the experimental unit. Data on absolute and relative STM and PM weight, absolute and relative organ weight, total area of STM, TNF of STM, P and S number of myofibers, the S:P ratio of STM_d_, area of STM_d_, STM_l_ and gene expression were analyzed including the main factors of IUC, DIET, sex, sow, parity, BtW (nested within sow), IUC × D and BtW × DIET interaction as fixed, and boar as random effect. For all analyses of the piglet data, sow was used as variable in a repeated measurement procedure. Birth weight was nested within sow as birth weight intervals (L and M) were not unique among sows, leading to the individual piglet being assigned either L or M depending on the dam. The final model was determined by performing backward elimination of non-significant three- and two-way interactions. Offspring was used as the observational unit.

Least squares means of the interaction means concerning the main factors IUC and DIET are presented in the result tables. As the effect of sex and BtW was rarely significant, these least square means were only reported in the text when *P* < 0.05. The PAIRWISE option using the Tukey adjustment was used to determine differences between interaction effects when two-way interactions occurred. Differences were considered statistically significant at *P* < 0.05 and as tendency at 0.05 < *P* < 0.10.

## Results

### Sow performance and litter characteristics

The number of total born and born alive piglets, and the male to female ratio were not different between IN and OL sows and were also not affected by the dietary ARG supply (Table 3). Average litter BtW, average BtW of female and male newborns as well as BtW variability, expressed as standard deviation (SD), also did not differ between IN and OL sows and between the ARG and Ctrl dietary treatments. Regarding stillborns, the five IN sows gave birth to one, two, two, four and five stillborn piglets, respectively, and only one OL sow gave birth to one stillborn piglet. Thus, the number of stillborn piglets were unevenly distributed: IN-Ctrl = 7; IN-ARG = 7; OL-Ctrl = 1; OL-ARG = 0. Due to the greatly unbalanced distribution between experimental treatment groups, statistical analysis of number and BtW of stillborn piglets was not performed.

**Table 3:** Reproductive characteristics of oviduct ligated (OL; non crowded) and intact (IN; relatively crowded) sows fed an unsupplemented (Ctrl) or l-arginine supplemented gestation diet (ARG) from d 14 to 28 of gestation^1^

	IN		OL			*P*-value^2^		
Item	Ctrl	ARG	Ctrl	ARG	SEM	IUC	DIET	IUC × DIET
Observations	5	5	5	5				
Total born	15.5	15.3	10.5	11.9	1.71	0.255	0.722	0.657
Born alive	13.1	14.5	9.1	10.3	1.49	0.230	0.463	0.961
Male:female ratio	0.47	0.62	0.50	0.41	0.093	0.508	0.732	0.368
Birth weight, kg								
Total born	1.22	1.48	1.57	1.51	0.068	0.278	0.214	0.126
Born alive								
All	1.24	1.50	1.59	1.53	0.068	0.277	0.224	0.133
Male	1.31	1.46	1.74	1.63	0.079	0.224	0.511	0.087
Female	1.26	1.51	1.50	1.44	0.092	0.580	0.365	0.230
SD^c^	0.24	0.25	0.18	0.14	0.056	0.414	0.869	0.626

^1^Results are presented as least squares means of interactions between the two main factors extent of intra uterine crowding and dietary treatment and pooled SEM

^2^Probability values for the effects of extent of intra uterine crowding (IUC), dietary treatment (DIET), and interaction IUC × DIET

^3^Measure for variability in birth weight, expressed as standard deviation (SD).

### Birth weight and absolute and relative muscle and organ weights

There were no significant effects of sow type and diet type on BtW of total born and born alive piglets (Table 3). Birth weight of males born alive from IN sows fed the ARG diet were heavier in tendency than those fed the Ctrl diet, whereas BtW of males born alive were lighter in tendency when originating from OL sows fed the ARG instead of the Ctrl diet (IUC × DIET interaction; *P* = 0.087). The average BtW of the selected offspring was lower than of the litter average because half of the piglets were of deliberately low BtW (Table 4). The piglets selected from the OL sows compared with those from the IN sows were heavier (*P* = 0.008) at birth, and had greater (*P* ≤ 0.033) STM, PM, liver and kidney weights. The weight of the spleen tended (*P* = 0.068) to be greater in OL compared with IN offspring. When expressed per 100 g BtW, the relative weights of the muscles and organs did not differ. The selected offspring from ARG compared with Ctrl sows tended (*P* < 0.10) to have greater BtW, absolute STM weight, liver weight and a lower brain:liver weight ratio. Except for the relative heart weight, which was lower (*P* = 0.030) in ARG than Ctrl offspring, muscle and organ weights relative to BtW were not affected by the dietary treatment of the sow. There was an IUC × DIET interaction (*P* = 0.005) indicating that absolute heart and relative spleen weight were greater and lower, respectively, in IN-ARG compared with IN-Ctrl piglets. Birth weight had no effect on either absolute or relative STM, PM, organ weights and brain:liver weight ratio. There were a number of significant (*P* < 0.05) diet × BtW interactions. Compared with L-piglets, M-piglets born from sows fed the ARG, but not the Ctrl diet, had heavier STM (L-ARG = 2.679 vs. M-ARG = 3.401 g), PM (L-ARG = 2.840 vs. M-ARG = 3.547 g), hearts (L-ARG = 8.387 vs. M-ARG = 9.538 g), livers (L-ARG = 33.87 vs. M-ARG = 41.07 g), spleens (L-ARG = 1.07 vs. M-ARG = 1.42 g), lungs (L-ARG = 17.00 vs. M-ARG = 19.90 g) and kidneys (L-ARG = 9.66 vs. M-ARG = 12.00 g). On the other hand, L-piglets compared with M-piglets born from sows fed the ARG had higher relative brain weights (L-ARG = 2.80 vs. M-ARG = 2.25) and a higher brain:liver weight ratio (L-ARG = 1.14 vs. M-ARG = 0.90) (DIET × BtW interaction; *P* < 0.001). No such differentiation was observed between L-Ctrl vs. M-Ctrl. It is also noteworthy that the absolute brain weight did not differ between L-ARG (33.5 g) and M-ARG (33.8 g). Together, these results suggest that besides the obvious positive association between BtW of piglet and organ size, an early gestational ARG supplementation has slightly greater effect on organ development of offspring with intermediate compared with low BtW. Finally, males and females differed (*P* < 0.05) in absolute weights of liver (34.18 vs. 36.73 g), lung weight (18.80 vs. 17.82 g) and relative heart weight (0.69 vs. 0.66 g/100 g BtW).

**Table 4:** Birth weight, absolute and relative muscle and organ weights and brain:liver weight ratio of offspring born from oviduct ligated (OL; non crowded) and intact (IN; relatively crowded) fed an unsupplemented (Ctrl) or l-arginine supplemented diet (ARG) from d 14 to 28 of gestation^1^

	IN		OL				*P*-value^2^			
Item	Ctrl	ARG	Ctrl	ARG	SEM	IUC	DIET	BtW	IUC×DIET	DIET×BtW
Observations	19	19	17	15						
Birth weight, kg	1.06	1.21	1.58	1.59	0.128	0.008	0.068	0.564	0.137	< 0.001
Weights, g										
*Semitendinosus* muscle	2.08	2.48	3.47	3.60	0.417	0.025	0.074	0.571	0.426	0.009
*Psoas major* muscle	2.15	2.36	3.86	4.02	0.343	0.001	0.232	0.543	0.891	0.024
Heart	8.34^a^	10.54^b^	8.35^ab^	7.38^ab^	1.695	0.418	0.159	0.806	0.005	0.032
Liver	20.91	27.39	45.98	47.55	5.808	0.004	0.053	0.529	0.290	0.022
Spleen	1.05	1.00	1.45	1.50	0.164	0.063	0.938	0.918	0.537	0.052
Lungs	16.48	18.24	19.86	18.66	2.655	0.576	0.750	0.459	0.152	0.010
Kidneys	7.68	8.93	12.77	12.73	1.516	0.033	0.301	0.771	0.322	0.022
Brain	32.22	33.01	33.85	34.32	0.781	0.235	0.119	0.621	0.699	0.031
Brain:liver weight ratio	1.36	1.12	0.94	0.92	0.222	0.270	0.077	0.151	0.182	0.001
Weights, g/100 g BtW										
*Semitendinosus* muscle	2.09	2.08	2.19	2.28	0.095	0.328	0.415	0.716	0.341	0.511
*Psoas major* muscle	2.06	2.14	2.31	2.39	0.140	0.277	0.276	0.314	0.987	0.483
Heart	0.70	0.67	0.67	0.65	0.023	0.492	0.030	0.770	0.720	0.338
Liver	2.21	2.47	2.78	2.77	0.179	0.128	0.192	0.629	0.142	0.343
Spleen	0.10^b^	0.09^a^	0.09^ab^	0.09^ab^	0.006	0.665	0.176	0.734	0.018	0.556
Lung	1.47	1.39	1.31	1.27	0.109	0.363	0.250	0.235	0.762	0.655
Kidney	0.74	0.72	0.80	0.82	0.043	0.270	0.764	0.613	0.396	0.627
Brain	2.87	2.53	2.47	2.50	0.323	0.616	0.169	0.204	0.114	< 0.001

^1^Results are presented as least squares means of interactions between the two main factors extent of intra uterine crowding and dietary treatment and pooled SEM

^2^Probability values for the effects of extent of intra uterine crowding (IUC), dietary treatment (DIET), birth weight (BtW), and interactions IUC × DIET and DIET × BtW

^a,b^Within a row for the main factor treatment, least squares means without a common superscript differ (*P* < 0.05)

### Myofiber characteristics of the *Semitendinosus* muscle

Except for the greater (*P* = 0.078) area of the STM_d_ in OL compared with IN offspring, the extent of IUC did not affect total muscle area, muscle area of the STM_l_ nor STM_d_ and STM_l_ myofiber number of selected offspring (Table 5). By contrast, the areas of the STM_d_ and STM_l_ and, consequently, of the whole STM were larger (*P* ≤ 0.025) in offspring born from ARG sows compared with Ctrl sows. However, regarding the STM_d_ area, ARG supplementation had a greater impact on OL compared to IN offspring (IUC × DIET; *P* = 0.007). In addition, the STM tended to be larger in M piglets from ARG sows compared with piglets from the other BtW categories (STM; M-ARG = 8.573; L-ARG = 7.002; M-Ctrl = 6.996; L-Ctrl = 6.271 μm^2^ × 10^7^, and STM_l_; M-ARG = 5.494; L-ARG = 4.301; M-Ctrl = 4.181; L-Ctrl = 3.875 μm^2^ × 10^7^; DIET × BtW interaction, *P* ≤ 0.061). Total myofiber number was 12% greater (*P* = 0.003) in the ARG group; this is primarily a result of a 13% greater (*P* = 0.022) TNF of the STM_l_. In the STM_d_, where differentiation between P and S myofibers was possible, the number of S myofibers, but not P myofibers, was numerically (*P* = 0.124) greater in piglets of ARG sows. As a consequence, the S:P myofiber ratio tended (*P* = 0.051) to be greater in the STM_d_ of these piglets. While L- and M-piglets did not differ with respect to total muscle area and TNF of the whole STM, BtW had an effect (*P* < 0.05) on TNF in the STM_d_ (L = 188,925 vs. M = 208,792), the number of S myofibers (L = 182,927 vs. M = 202,776) and tended to have an effect (*P* = 0.069) on the number of P myofibers (L = 5,998 vs. M = 5,986) also in the STM_d_. Due to the lack of an effect of BtW and of a DIET × BtW interaction in TNF, the positive effect of ARG supplementation on myofiber hyperplasia was similar for L and M piglets (L = 506,274 vs. M = 567,783 myofibers).

**Table 5:** Myofiber characteristics in the *Semitendinosus* muscle of selected offspring born from oviduct ligated (OL; non crowded) and intact (IN; relatively crowded) sows fed an unsupplemented (Ctrl) or l-arginine supplemented diet (ARG) from d 14 to 28 of gestation^1^

	IN		OL				*P*-value^2^			
Item	Ctrl	ARG	Ctrl	ARG	SEM	IUC	DIET	BtW	IUC×DIET	DIET×BtW
Observations	19	19	17	15						
Muscle area, μm^2^ × 10^7^										
Total	5.762	6.780	7.505	8.795	0.9429	0.144	0.003	0.653	0.738	0.053
Dark portion	2.359^x^	2.278^x^	2.772^ax^	3.572^by^	0.2931	0.078	0.025	0.233	0.007	0.573
Light portion	3.151	4.118	4.906	5.677	0.8279	0.143	0.011	0.910	0.786	0.061
Myofiber number, N										
Total muscle	476,025	527,711	536,451	607,928	35,511.0	0.236	0.003	0.136	0.604	0.475
Dark portion										
Total myofibers	193,928	198,254	184,590	218,604	22,898.2	0.884	0.131	0.002	0.231	0.560
Primary myofibers (P)	6,105	5,840	5,868	6,156	638.8	0.970	0.974	0.069	0.422	0.575
Secondary myofibers (S)	187,823	192,414	178,722	212,448	22,455.9	0.883	0.124	0.002	0.231	0.559
S:P ratio^3^	30.8	33.2	30.6	34.4	2.86	0.912	0.051	0.356	0.643	0.337
Light portion	282,097	329,457	351,860	389,324	32,639.6	0.234	0.022	0.889	0.778	0.236

^1^Results are presented as least squares means of interactions between the two main factors extent of intra uterine crowding and dietary treatment and pooled SEM

^2^Probability values for the effects of extent of intra uterine crowding (IUC), dietary treatment (DIET), birth weight (BtW), and interactions IUC × DIET and DIET × BtW

^3^The secondary-to-primary myofiber ratio, calculated by dividing the number of secondary with the number of primary myofibers in the dark portion of the *Semitendinosus* muscle.

^a,b^Within a row for the main factor treatment, least squares means without a common superscript differ (*P* < 0.05)

^x,y^Within a row for the main factor treatment, least squares means without a common superscript differ (0.05 < *P* < 0.10)

### Gene expression in the *Semitendinosus* muscle

The level of *PRKAA2* expression in the STM was greater (*P* = 0.024) in IN compared with OL offspring (Table 6). In addition, ARG appeared to have a greater impact on the expression of this gene in IN than in OL offspring (IUC × DIET; *P* = 0.034). Furthermore, a IUC × DIET interaction effect (*P* = 0.013) was found in the expression of *IGF2*; however, Tukey's *post hoc* comparison did not identify individual differences between means. The expression of *MYOD1* tended (*P* = 0.056) to be lower in L- than M-pigs (L = 1.96 vs. M = 1.86), and a tendency (*P* = 0.091) to a DIET × BtW interaction effect on *PRKAA2* expression was found, where M-piglets from Ctrl sows displayed the lowest expression compared with the remaining piglets (M-Ctrl = 0.44; L-Ctrl = 0.53; M-ARG = 0.59; L-ARG = 0.60 relative expression level). Again, there were no differences in individual interaction means identified by the Tukey's *post hoc* comparison.

**Table 6:** Relative expression of myogenesis-related genes in the *Semitendinosus* muscle of selected offspring born from oviduct ligated (OL; non crowded) and intact (IN; relatively crowded) sows fed an unsupplemented (Ctrl) or l-arginine supplemented diet (ARG) from d 14 to 28 of gestation^1^

	IN		OL			*P*-value^2^				
Gene^3^	Ctrl	ARG	Ctrl	ARG	SEM	IUC	DIET	BtW	IUC×DIET	DIET×BtW
Observations	16	17	17	15						
*IGF2*	2.36	1.55	0.91	1.70	0.555	0.487	0.967	0.965	0.013	0.950
*IGFBP5*	5.63	5.65	2.32	4.62	2.190	0.489	0.233	0.275	0.290	0.297
*MSTN* ^4^	1.60	1.64	0.46	0.98	1.754	0.351	0.232	0.105	0.241	0.110
*MYF5* ^4^	0.35	0.42	0.24	0.28	1.694	0.631	0.531	0.895	0.974	0.159
*MYF6*	0.58	0.65	1.18	0.98	0.576	0.623	0.859	0.893	0.664	0.631
*MYOD1* ^4^	2.36	1.84	0.68	0.48	2.143	0.223	0.340	0.056	0.885	0.418
*MYOG*	0.94	0.70	1.32	1.11	0.255	0.333	0.168	0.392	0.915	0.417
*PRKAA2*	0.72^x^	1.04^by^	0.26^x^	0.15^ax^	0.194	0.024	0.264	0.298	0.034	0.091

^1^Results are presented as least squares means of interactions between the two main factors extent of intra uterine crowding and dietary treatment and pooled SEM.

^2^Probability values for the effects of extent of intra uterine crowding (IUC), dietary treatment (DIET), birth weight (BtW), and interactions IUC × DIET and DIET × BtW

^3^ *IGF2* Insulin-like growth factor 2, *IGFBP5* Insulin-like growth factor binding protein 5, *MSTN* Myostatin, *MYF5* myogenic factor 5, *MYF6* myogenic factor 6, *MYOD1* Myogenic differentiation 1, *MYOG* Myogenin (myogenic factor 4), *PRKAA2* Protein kinase AMP-activated, alpha 2 catalytic subunit

^4^Statistical evaluation was performed with log transformed data

^a,b^Within a row for the main factor treatment, least squares means without a common superscript differ (*P* < 0.05)

^x,y^Within a row for the main factor treatment, least squares means without a common superscript differ (0.05 < *P* < 0.10)

### Discussion

The increased litter size of modern sow breeds is likely associated with increased incidents of IUC and consequently IUGR. Therefore, an increased proportion of underdeveloped piglets within the litters can be observed [21, 22]. These piglets display a lower postnatal survival rate [23], and a poorer growth performance compared with their larger littermates [24, 25]. These impairments can in part be attributed to the reduced myofiber hyperplasia mainly due to lower formation of S myofibers [8]. One approach to enhance hyperplasia is by supplementing l-arginine to the sows during early gestation, shown to be leading to increased formation of P myofibers, which serve as a scaffold for the development of the S myofibers [13]. To alter the intra-uterine environment, either unilateral ovary-hysterectomy or unilateral oviduct ligation of sows can be applied, where sows subjected to the latter procedure have shown to display minimal crowding and adequate placental development [18]. Thus, compared with intact natural crowded sows as used in the current study, OL sows with average prolificacy are suitable models for investigating the consequences of IUC and IUGR in newborn piglets. Hence, intact prolific sows with naturally crowded uterus and OL sows with a “non-crowded uterus” were used in the present study to mimic two degrees of IUC and to investigate whether the positive effect of early gestational supplementation of the sows with l-arginine on myofiber hyperplasia observed in 75-day-old fetuses [13] would continue until birth and would differ depending on the extent of IUC.

### Sow performance and litter characteristics

Important causes of IUGR are impaired placental development, including suboptimal vascularization of the placenta, increased numbers of fetuses due to high prolificacy and uneven distribution of energy and dietary nutrients among the fetuses [26]. As indicated by the number of total and born alive piglets in combination with the corresponding BtW, IUGR occurred to a greater extent in IN sows compared with OL sows. A sow with a litter size of ≥ 15 is categorized as being high prolific [24, 27]. This was the case with the IN sows. On the contrary, OL sows had a litter size below the average herd level (9.7 vs. 12.3 live born). The OL sows compared with the IN sows gave birth to a smaller number of stillborn piglets, which was expected from previous findings associating increased litter size with a greater number of stillborn piglets [27]. Litter characteristics of IN sows were in agreement with results from a previous study where offspring developing in a crowded compared with an uncrowded intra-uterine environment exhibited phenotypes associated with IUGR [18]. In the present study, performance of OL sows with respect to litter size and average BtW was comparable to that observed in low to average prolific sows [22].

Effects of maternal dietary l-arginine supplementation on offspring have been previously investigated, but both dietary level, onset of supplementation during gestation and impact on sow and offspring traits have varied greatly among different studies as reviewed by Wu et al. [28]. Among reported effects of l-arginine are increased numbers of total born, born alive and total litter BtW in multiparous sows [29]. However, the sows included in the two former studies had an average litter size of 13 to 14 piglets, thus are to be categorized as average rather than highly prolific sows with no expected major impact of IUC and IUGR. In the present study, dietary supplementation of l-arginine did not significantly affect the sow reproductive performance. Nevertheless, numerical improvements of particularly the number of piglets born alive as well as a greater BtW of offspring in IN-ARG compared with IN-Ctrl sows were observed. However, the reproduction data must be interpreted with caution due to the low number of sows used in this study.

### Muscle and organ weights of offspring

The greater BtW and weight of STM and PM of offspring from OL sows compared with IN sows were foreseen as it has been reviewed extensively that there is a high correlation between large litter size and extent of crowding as well as greater within-litter variation of BtW and greater number of low BtW offspring [2, 30, 31]. Organ weights of offspring were within the normal range of newborn piglets [18, 32]. The tendency for an increased absolute STM weight in offspring from ARG sows suggests that, independent of IUC, l-arginine supplementation promotes muscle development of offspring, an observation that has to the best of the authors’ knowledge not been shown in previous studies.

Besides reduced BtW, brain:liver weight ratio has been shown to positively correlate with uterine crowding [11] and therefore is an indicator of IUGR [33]. The brain-sparing mechanism ensures steady relative brain weight development when maternal nutrient supply does not match requirements for adequate fetal development [34]. The greater liver weight combined with the similar brain weight resulted in a lower brain:liver weight ratio in offspring from ARG sows compared with Ctrl sows and supports the hypothesis that l-arginine supplementation has the potential to alleviate the negative impact of IUGR. In addition, the absolute heart weight was only numerically greater in ARG compared with Ctrl, an observation that has also been related to low birth weight pigs at slaughter age [35]. Thus, with the exception of the brain:liver ratio, this observation would not support the positive effect of l-arginine on organ development under crowding conditions.

Due to the rather high level and intake of crude protein and supplementation of daily l-arginine, a great load of maternal arginine could be expected. However, as discussed above, no detrimental effects on either the sow or the offspring traits were observed, indicating extensive catabolism of arginine and that maternal intake of arginine was not excessive [36]. Thus, it was concluded that the level of supplemented arginine was within the safety margin eliciting no toxicity or adverse effects [37, 38], whereas higher crude protein level and arginine concentration of the diet might lead to impaired reproductive performance, antagonism between amino acids and toxicity of ammonia to sows and their fetuses [28].

### Myofiber-related traits of the *Semitendinosus* muscle

It is widely accepted that the formation of S myofibers is highly dependent on fetal nutrient uptake [12]. Thus, a reduced nutrient availability in the placenta during mid to late gestation, is likely to result in a lower S myofiber hyperplasia. Previous studies showed that a lower number of myofibers was associated with reduced muscle mass at weaning and at slaughter age [6, 39] meaning an ongoing disadvantage during the entire fattening performance. In the present study, supplementing l-arginine to the sows during early gestation resulted in an increased TNF, with the greatest contribution derived from the second wave of myofiber formation as indicated by the greater S:P ratio in the STM_d_ and the greater TNF in the STM_l_. In comparison, a similar previous study reported that early gestational supplementation with l-arginine enhanced prenatal hyperplasia, but, in contrast, the main increase of TNF originated from the formation of P myofibers [13]. However, in that study myofiber hyperplasia was assessed at d 75 of gestation, a time point where hyperplasia of S myofibers is still ongoing. It is worth mentioning that the increased hyperplasia observed in offspring from ARG sows was independent of BtW (L-ARG: 529,186 vs. M-ARG: 606,453; *P* = 0.136). This finding contradicts our hypothesis that underprivileged fetuses would preferentially benefit from ARG supplementation by an increased placental vascularization. Results of a recent study [40] suggested that supplementing the gestational diet with either l-arginine, ractopamine, or a combination of the two increased myofiber diameter, indicating that supplementation had an effect on muscle fiber hypertrophy rather than hyperplasia. Ultimately, this resulted in a lower proportion of low BtW piglets (< 0.8–1.2 kg BtW), and an increased proportion of medium and high BtW piglets (> 1.2–1.6 kg BtW) from l-arginine supplemented compared with unsupplemented sows [40]. However, interestingly and in contrast to the present study, a lower TNF was observed in offspring from supplemented compared with unsupplemented sows. One explanation for the discrepancy between the observation in our study and the study of Garbossa et al. [40] could be the mode of l-arginine supplementation, either during d 14-28 or 25-53 of gestation, respectively, and at either 0.89% or 1.00% of the diet, respectively. With these combined results in mind, it is relevant to reflect on the extent to which supplementary l-arginine is directing dietary energy for hyperplasia or hypertrophy in fetuses during the different phases of gestation. Furthermore, investigating the impact of gestational supplementation on hyperplasia especially in post-weaning pigs would be of great importance, as results on this matter are contradictory. For instance, supplementing l-carnitine daily to L-BtW piglets during the suckling period resulted in a third wave of hyperplasia [41] during the nursing period, but no difference in TNF was found between control and l-carnitine supplemented pigs at the age of slaughter [42]. Based on this contradiction, one could speculate that changes occurring during the prenatal period are more manifested and less prone to environmental changes in the postnatal period, thus, having permanent positive effects until the animal reaches slaughter weight.

In light of this discussion and based on earlier findings [43, 44], one can assume that maternal arginine concentration was increased in plasma of sows fed l-arginine during the gestational period from d 14-28.

### Gene expression of myogenesis-related genes in the *Semitendinosus* muscle

Muscle development during the perinatal period requires the regulation of several myogenesis related genes [45]. In the present study none of the genes analyzed were responding to the extent of IUC or dietary treatment, except for *PRKAA2*, the expression of which was up-regulated in the STM of IN offspring compared with OL offspring. *PRKAA2* is a known inhibitor of muscle protein synthesis [46], and its greater expression in IN offspring is consistent with their lower BtW and relative STM weight emphasizing the importance of this gene in pre-natal muscle development. In contrast to the increased TNF at birth elicited by maternal l-arginine supplementation, results from the gene expression analysis did not support a myogenic effect at the molecular level. As hyperplasia ceases around d 90 of gestation, genes involved in myogenesis including myofiber formation might no longer show a specific up-regulation, but would rather be expressed at a steady state at birth when the STM was sampled. However, although there was no direct effect of l-arginine supplementation on the gene expression of myogenesis-related genes, it appears that *IGF*2 and *PRKAA2* expressions in neonate STM could be influenced by the dietary level of this particular amino acid in certain uterine environments because there was a significant interaction.

## Conclusion

In conclusion, the present study further adds to the increasing evidence that intra-uterine crowding prevalent in high prolific sows critically affects the phenotype of the offspring. This was manifested by the low BtW in contrast to the moderate litter size and offspring BtW of the OL counterparts. Confirming our first hypothesis, l-arginine in the early gestational diet seems to reduce the negative impacts of IUGR, as shown by the increased hyperplasia, body weight and STM area of the offspring at birth. As muscle area increased more than TNF, l-arginine supplementation obviously not only enhanced prenatal myofiber hyperplasia but also hypertrophy. However, details concerning the way in which l-arginine affects myofiber development on cellular and molecular levels remain to be determined. The second hypothesis that l-arginine would be especially efficient in L-BtW piglets was not confirmed. Still, this feeding strategy could be of great benefit to especially L-BtW pigs as they are particularly vulnerable. Thus, l-arginine supplementation to sows in early gestation would potentially improve the survival rate during nursery, and the growth potential during fattening of L-BtW piglets.

## Abbreviations

ARG: Arginine supplementation
BtW: Birth weight
Ctrl: Alanine supplementation
DIET: Dietary treatment
IGF2: Insulin-like growth factor 2
IGFBP5: Insulin-like growth factor binding protein 5
IN: Intact sows
IUC: Intra-uterine crowding
IUGR: Intra-uterine growth retardation
L: Low
M: Intermediate
MSTN: Myostatin
MYF5: Myogenic factor 5
MYF6: Myogenic factor 6
MYOD1: Myogenic differentiation 1
MYOG: Myogenin (myogenic factor 4)
OL: Unilateral oviduct ligated
P: Primary
PM: *Psoas major*
PRKAA2: Protein kinase AMP-activated alpha 2 catalytic subunit
RPL4 (LOC100038029): Ribosomal protein L4
S: Secondary
SD: Standard deviation;
STM: *Semitendinosus* muscle;
STM_d_: *Semitendinosus* muscle dark portion
STM_l_: *Semitendinosus* muscle light portion
TBP: TATA box binding protein TNF, total number of myofibers
